# A Rare Clinical Presentation of Metastatic Crohn’s Disease

**DOI:** 10.7759/cureus.8285

**Published:** 2020-05-26

**Authors:** Kaitlyn L Streight, Tara L Braun, Nicholas Lowe, Soo Jung Kim

**Affiliations:** 1 Dermatology, Baylor College of Medicine, Houston, USA

**Keywords:** metastatic crohn’s disease, cutaneous crohn’s disease, cutaneous manifestations of systemic disease, crohn’s disease

## Abstract

A 31-year-old female with a history of systemic lupus erythematous, IgA nephropathy, and psoriasis presented with a one-month history of a painful, pruritic rash under the bilateral breasts and in the genital region. Cutaneous examination revealed a large, tender ulcer under the left breast with a shiny erythematous base and peripheral hypertrophic changes. Small ulcers were present on the bilateral inguinal folds, and the labia majora were edematous with multiple erythematous papules. Histological examination of the left breast revealed ulceration with granulomatous dermatitis, consistent with a diagnosis of metastatic Crohn’s disease. Metastatic Crohn’s disease is a rare cutaneous manifestation of Crohn’s disease characterized by non-caseating granulomas in regions non-contiguous with the gastrointestinal tract. At the time of diagnosis, our patient reported no gastrointestinal symptoms aside from occasional blood-streaked stools from hemorrhoids. This case demonstrates the importance of considering the disease when a patient presents with intertriginous or genital lesions, even in the absence of systemic manifestations.

## Introduction

Crohn’s disease is a subtype of inflammatory bowel disease characterized by segmental, granulomatous lesions of the intestinal tract [1]. Cutaneous manifestations are common and typically occur in regions contiguous with the gastrointestinal tract, such as the perianal and oral region. Metastatic Crohn’s disease (MCD), in contrast, is a rare cutaneous manifestation of Crohn’s disease characterized histologically by non-caseating granulomas in regions non-contiguous with the gastrointestinal tract [1-3]. While most patients with MCD carry a previous diagnosis of Crohn’s disease, some patients present without classic gastrointestinal manifestations [4]. The clinical characteristics of MCD also vary, suggesting that the disease may be underrecognized due to misdiagnosis [5]. Herein, we report a rare presentation of MCD with involvement of the breast and genital regions in the absence of active intestinal manifestations.

## Case presentation

A 31-year-old female with a history of systemic lupus erythematous, IgA nephropathy, and psoriasis presented with a painful, pruritic rash involving the inframammary and genital skin. The rash appeared approximately one month prior and progressively worsened since its onset. The patient stated that she applied topical hydrocortisone under her breasts without relief. She denied any diarrhea, hematochezia, or abdominal pain but admitted to occasional blood streaked stools from hemorrhoids. Colonoscopy revealed rectal ulceration and exam-limiting stricture.

**Figure 1:**
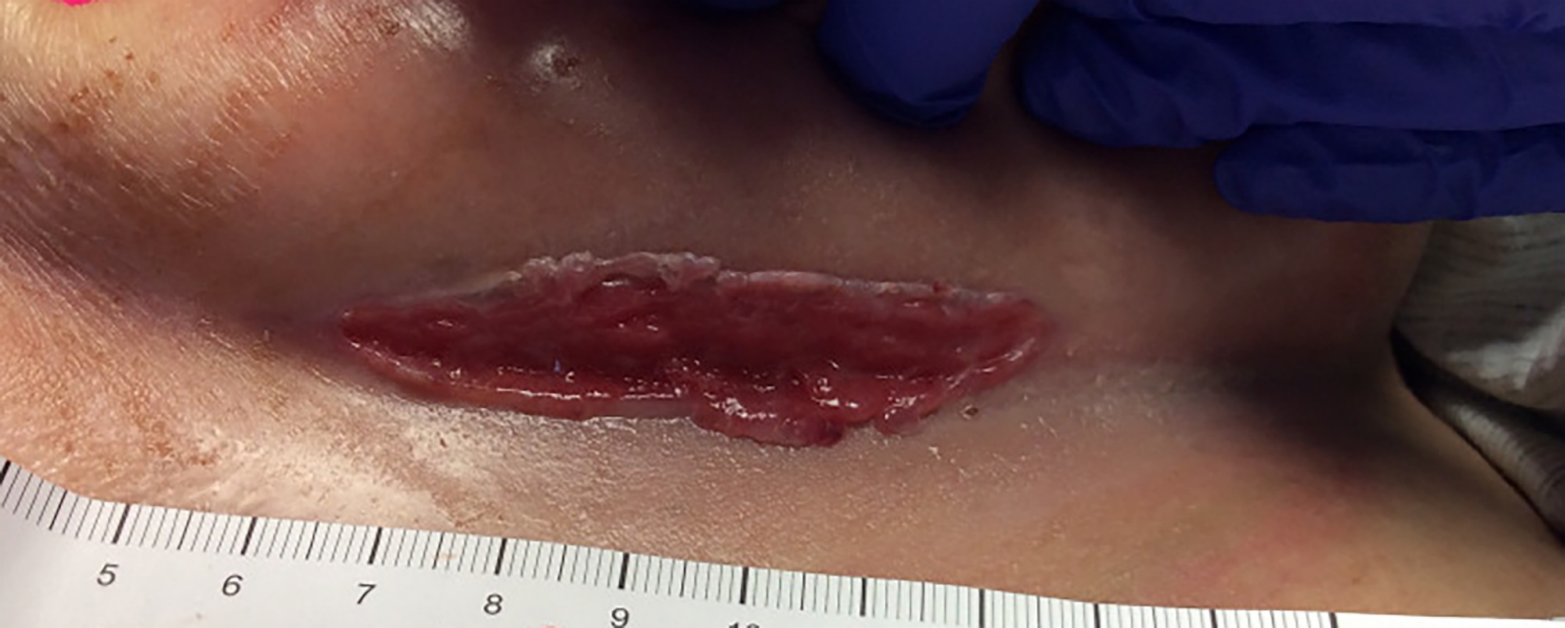
Left inframammary fold Large, 6-cm ulcer with a shiny, erythematous base.

Cutaneous examination revealed a large, tender ulcer under the left breast with a shiny erythematous base and peripheral hypertrophic changes (Figure 1).

**Figure 2:**
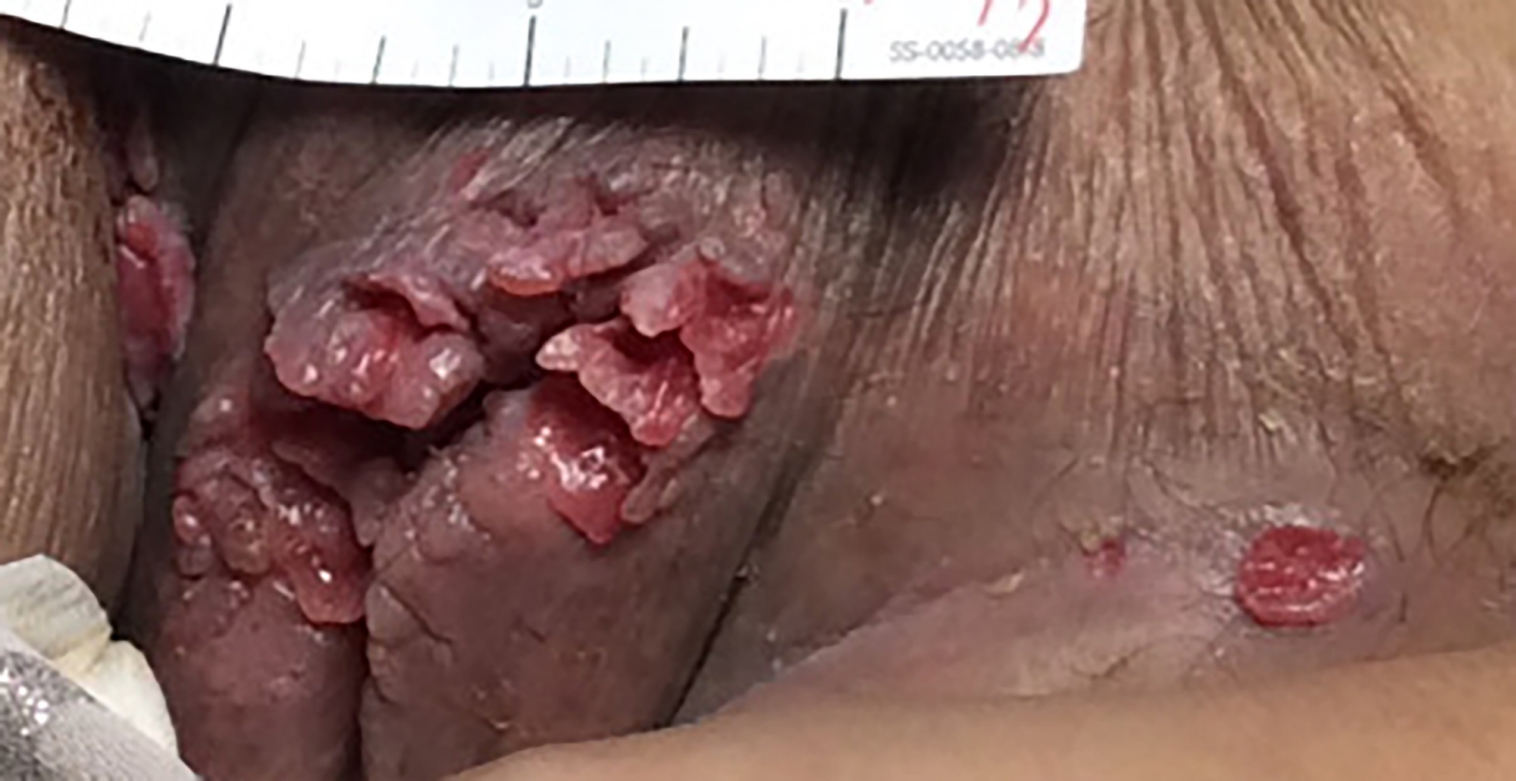
Groin region Edematous labia majora with multiple verrucous, erythematous papules, and a 1-cm ulcer on the left inguinal crease.

An erythematous patch without ulceration was present under the right breast. There were small ulcers on the bilateral inguinal folds and multiple verrucous, erythematous, and skin-colored papules on the labia majora with edema (Figure 2). 

**Figure 3:**
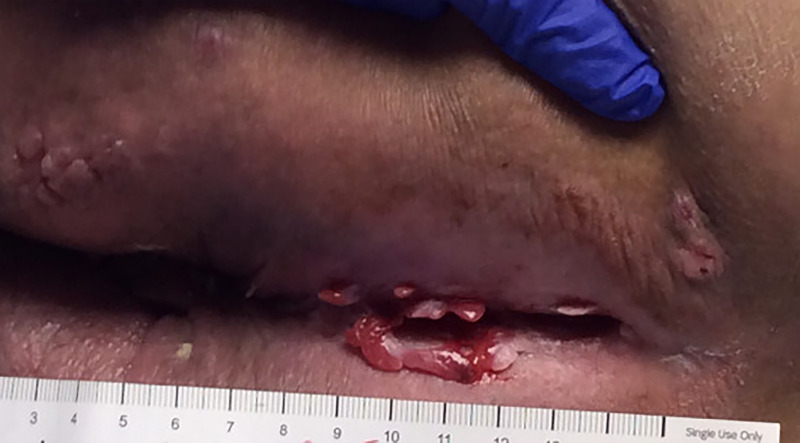
Intergluteal folds Multiple erythematous, shiny papules with interspersed fissuring.

Multiple papules with interspersed fissures were found between the intergluteal folds (Figure 3). Tangential biopsy of the left breast showed ulceration with granulomatous dermatitis, consistent with a diagnosis of MCD. 

## Discussion

Crohn’s disease is an inflammatory disease characterized by segmental, granulomatous lesions of the intestinal tract. Cutaneous manifestations occur in approximately 44% of patients and are confirmed by the histopathology, with characteristic non-caseating granulomas similar to the intestinal lesions seen in the disease [1-3]. Reactive lesions do not share the same histopathological findings and include manifestations such as pyoderma gangrenosum, erythema nodosum, and oral apthae [1,2]. Cutaneous Crohn’s disease manifests most commonly as specific lesions involving regions contiguous with the gastrointestinal tract, including perianal fissures or fistulae, peristomal fissures or fistulae, and oral lesions [3]. In contrast, MCD is an exceedingly rare dermatologic manifestation at cutaneous sites distinct from the gastrointestinal tract [4]. Due to the variable clinical presentation of MCD, many authors believe that the disease is underrecognized and frequently misdiagnosed [5].

MCD typically affects adults between the ages of 29 and 39 years, but reports have included all age groups [3,4]. Approximately 70%-90% of patients present with a prior diagnosis of intestinal Crohn’s disease, but in 10%-30% of cases the gastrointestinal tracts are not involved, as demonstrated by this case [6]. While there is no clear correlation between the development of MCD and the severity of Crohn’s disease, cases are more commonly seen in association with colonic lesions compared to lesions of the small bowel [7].

The morphologic characteristics of MCD vary depending on the location of lesions but often present as erythematous plaques, nodules, or ulcers most commonly on the legs, vulva, penis, trunk, and face [3,7]. A predilection for intertriginous areas has also been reported, as demonstrated by our case [3,8]. Genital involvement is more common in children and typically presents with ulceration, fissures, edema, and erythema [3,5,6]. Our patient presented with papules on the labial region, which has only been reported rarely in adults [3,5].

Histologically, MCD presents similarly to the gastrointestinal lesions of Crohn’s disease, with non-caseating granulomas in the superficial papillary and deep reticular dermis and occasional extension into the subcutaneous fat [3,4]. These granulomas consist of epithelioid and multinucleated histiocytes with a lymphocytic infiltrate, occasionally surrounding blood vessels in a phenomenon called granulomatous perivasculitis. While plasma cells and eosinophils may be present, neutrophils and focal necrobiosis are generally absent [3].

The differential diagnosis of MCD is broad due to the variable clinical presentation. When involving intertriginous regions, as in our patient, MCD can resemble hidradenitis suppurativa, seborrheic dermatitis, or intertrigo. On the limbs, other diagnoses to consider include cellulitis, allergic contact dermatitis, pyoderma gangrenosum, Wegener's granulomatosis, and eczematous dermatitis [3]. These entities can all be distinguished from MCD microscopically. On histology, the differential diagnosis includes other granulomatous entities, such as cutaneous sarcoidosis, erythema nodosum, pyoderma gangrenosum, hidradenitis suppurativa, mycobacterial diseases, and foreign body reactions [4].

Due to the rarity of MCD, there is currently no established treatment. Although spontaneous resolution has been reported, most cases persist without treatment [7]. Previously employed therapies with mixed results include topical steroids, topical calcineurin inhibitors, oral antibiotics (metronidazole) for mild and limited disease, oral steroids, azathioprine, sulfasalazine, 6-mercaptopurine, and infliximab for severe cases. Surgical excision with debridement has also shown success in cases refractory to other therapies [3,4]. With any treatment modality, recurrence may occur [7].

## Conclusions

Although rare, this case demonstrates the importance of considering MCD when a patient presents with intertriginous or genital lesions, even in the absence of active intestinal disease.
